# Dependencies of microstructure and stress on the thickness of GdBa_2_Cu_3_O_7 − *δ*_ thin films fabricated by RF sputtering

**DOI:** 10.1186/1556-276X-8-304

**Published:** 2013-07-01

**Authors:** Ying Wang, Da Xu, Yijie Li, Linfei Liu

**Affiliations:** 1 Key Laboratory of Artificial Structure and Quantum Control, Ministry of Education, Department of Physics, Shanghai Jiao Tong University, 800# Dong Chuan Road, Shanghai 200240, People’s Republic of China; 2 Department of Physics and Mathematics, Shanghai University of Electric Power, 28# Xue Hai Road, Shanghai 201300, People’s Republic of China

**Keywords:** Stress, Thickness dependencies, Superconducting performances

## Abstract

GdBa_2_Cu_3_O_7 − *δ*_ (GdBCO) films with different thicknesses from 200 to 2,100 nm are deposited on CeO_2_/yttria-stabilized zirconia (YSZ)/CeO_2_-buffered Ni-W substrates by radio-frequency magnetron sputtering. Both the X-ray diffraction and scanning electron microscopy analyses reveal that the *a*-axis grains appear at the upper layers of the films when the thickness reaches to 1,030 nm. The X-ray photoelectron spectroscopy measurement implies that the oxygen content is insufficient in upper layers beyond 1,030 nm for a thicker film. The Williamson-Hall method is used to observe the variation of film stress with increasing thickness of our films. It is found that the highest residual stresses exist in the thinnest film, while the lowest residual stresses exist in the 1,030-nm-thick film. With further increasing film thickness, the film residual stresses increase again. However, the critical current (*I*_c_) of the GdBCO film first shows a nearly linear increase and then shows a more slowly enhancing to a final stagnation as film thickness increases from 200 to 1,030 nm and then to 2,100 nm. It is concluded that the roughness and stress are not the main reasons which cause the slow or no increase in *I*_c_. Also, the thickness dependency of GdBa_2_Cu_3_O_7 − *δ*_ films on the *I*_c_ is attributed to three main factors: *a*-axis grains, gaps between *a*-axis grains, and oxygen deficiency for the upper layers of a thick film.

## Background

Second-generation high-temperature superconducting (HTS) coated conductors based on ReBa_2_Cu_3_O_7 − *δ*_ (REBCO, RE = Y, Gd, Sm, etc., rare earths) films are coming into practical applications for motors, fault current limiters, generators, and transformers [1,2]. High critical current (*I*_c_) is needed for many HTS applications. Apparently, enhancing the thickness of (RE) BCO films is the simplest method. However, there is an obstacle for this way as there is a current density (*J*_c_) decreasing phenomenon as films become thicker [3]. Such a falloff of *J*_c_ is found in ReBa_2_Cu_3_O_7 − *δ*_ films fabricated by different methods, such as pulsed laser deposition [4], hybrid liquid-phase epitaxy [5], Ba-F-based methods [6], and chemical solution deposition by ink-jet printing [7].

Several possible reasons for the so-called ‘thickness effect’ of *J*_c_ have been advanced. These include *a*-axis growth, the increase in surface roughness, and porosity. Another reasonable interpretation of the thickness effect of *J*_c_ has been proposed by Foltyn et al. [8]. They attributed this to misfit dislocations near the interface between the superconductor and the substrate. The same research group reported that by inserting several thin CeO_2_ layers, the thickness effect can be overcome in some extent [9]. The suppressed thickness effect may be due to much more interfaces between the superconductor and the substrate in the multilayer compared with the single layer. Variations of stress also may be an important factor for *J*_c_ to decrease with thickness. Xiong et al. [10] reported that variations of stress in yttrium barium copper oxide (YBCO) film resulted in first the increase and then the decrease of *J*_c_ with increasing film thickness. Similar results are found by Zeng et al. [11]. Many groups have made their efforts to find methods to eliminate the thickness effect of *J*_c_ with enhancing film thickness. However, a much deeper understanding of the development of residual stress and microstructure in ReBa_2_Cu_3_O_7 − *δ*_ films with different thicknesses is desired for the optimization of superconducting performance.

In the present work, GdBa_2_Cu_3_O_7 − *δ*_ (GdBCO) films with different thicknesses are fabricated by radio-frequency magnetron sputtering (RF sputtering) in order to understand the problems mentioned above, particularly with respect to microstructure and residual stress. X-ray diffraction (XRD), scanning electron microscopy (SEM), atomic force microscopy (AFM), and X-ray photoelectron spectroscopy (XPS) are performed to observe the texture, surface morphology, and oxygen content of GdBCO films. Meanwhile, the Williamson-Hall method is applied to calculate the residual stress in the studied films.

## Methods

Biaxially textured Ni-5 at.% W alloy tapes from EVICO GmbH (Dresden, Germany) are used in these studies. The out-of-plane and in-plane texture are 6° and 7°, respectively. The thickness of the alloy tape is 70 μm, and the width is 10 mm. The root mean square roughness (RMS) is no more than 7 nm over a 50 μm × 50 μm area. CeO_2_, yttria-stabilized zirconia (YSZ), and CeO_2_ films are in sequence fabricated on Ni-W tapes by RF sputtering. Firstly, CeO_2_ is fabricated. The formed gas Ar (97%) + H_2_ (3%) served as the sputtering gas to prevent the oxidation of alloy tapes. The total pressure is 0.02 Pa.

After the fabrication of the CeO_2_ seed layer, a total pressure of O/Ar mixture gas of 30 Pa is introduced to the chamber. Then the YSZ layer is fabricated. The YSZ (8% ZO_2_) target is used in the experiment. The sputtering power is 40 and 50 W for the CeO_2_ seed layer and the YSZ layer, respectively. The growth temperature is 760°C for both the CeO_2_ seed layer and the YSZ layer. The substrate-target distance is about 50 mm for both the CeO_2_ seed layer and the YSZ layer. The fabrication time is 30 min for the CeO_2_ seed layer and 60 min for the YSZ layer. Secondly, the CeO_2_ cap layer is fabricated. The parameters for the CeO_2_ cap layer are identical to those for the CeO_2_ seed layer. The O/Ar ratio is 1:5 for both the YSZ layer and the CeO_2_ cap layer. The thicknesses of the CeO_2_ seed layer, the YSZ layer, and the CeO_2_ cap layer are about 30, 70, and 30 nm, respectively.

The microstructure features of CeO_2_/YSZ/CeO_2_-buffered Ni-W substrates are measured. The out-of-plane and in-plane are 4.3° and 7.0°, respectively. The AFM image shows a smooth and no-crack surface morphology of the CeO_2_ cap layer. At last, the GdBCO films are fabricated on CeO_2_/YSZ/CeO_2_-buffered Ni-W substrates by RF sputtering. During the GdBCO film fabrication, the substrate temperature, O/Ar mixed gas pressure, and sputtering power are 780°C, 25 Pa, and 80 W, respectively. The O/Ar is 1:1.

Seven samples with various thicknesses are fabricated. Film thickness is controlled by different sputtering times, while other parameters are fixed. The thickness for the studied samples is measured using a step profiler. The seven samples are 5 cm long and 1 cm wide. In order to get an average thickness of our samples, especially for the thicker films with *a*-axis outgrowths, ten points along the sample width direction are chosen for thickness measurement using the step profiler for every sample. The distance between the chosen points is 0.1 cm. The average thicknesses of our samples are 200, 390, 602, 810, 1,030, 1,450, and 2,100 nm, respectively. The thickness homogeneity along the length direction (not the width direction) is very good for the studied samples. Four films are used to analyze the development of the microstructure and stress of GdBCO films. Their thicknesses are 200, 1,030 1,450, and 2,100 nm, and they are named F200, F1030, F1450, and F2100, respectively. The microstructure and stress of the films are studied by XRD, SEM, AFM, and XPS analysis. The *I*_c_ is measured using the standard four-probe method. A voltage criterion of 1 μV/cm is used to determine *I*_c_ in the *I*-*V* curves.

## Results and discussion

### Film texture and surface morphology

**Figure 1:**
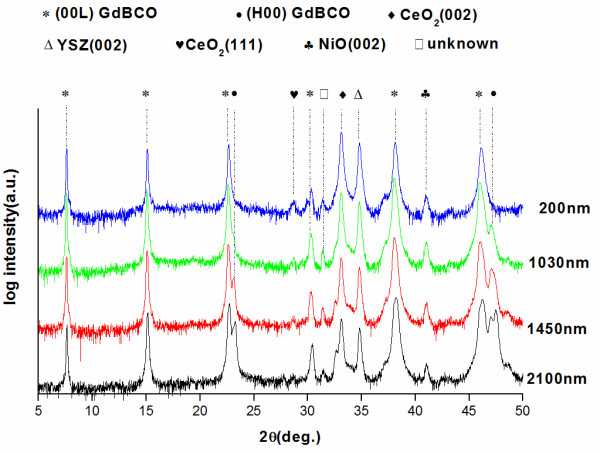
X-ray diffraction patterns for the GdBCO films with different thicknesses.

**Figure 2:**
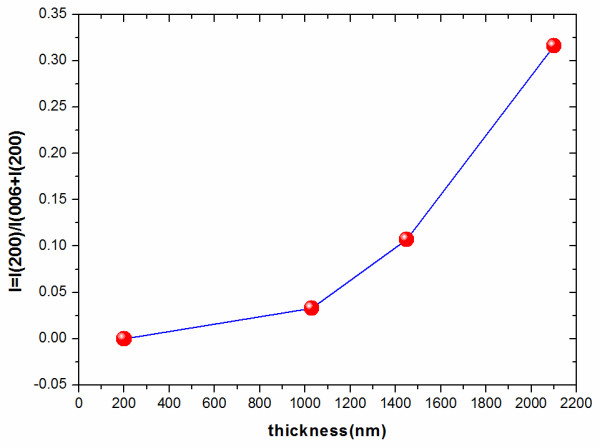
**The thickness dependency of the relative ratio of the content of *a*-axis grains versus *c*-axis grains.**

Figure 1 shows the log scale of *θ*-2*θ* XRD patterns for the GdBCO films with different thicknesses from 200 to 2,100 nm. Except for the peaks from the CeO_2_/YSZ/CeO_2_-buffered Ni-W substrate and other three small peaks, all of the peaks can be attributed to GdBCO films. Weak CeO_2_ (111) and NiO (002) peaks appear at 28° and 41°, respectively. The weak CeO_2_ (111) peak originates from the buffer layers, while the NiO (002) peak suggests that there is a minor oxidation of the Ni-W substrate. The (00*L*) peaks belong to *c*-axis grains. The (*H*00) peaks indicate *a*-axis grains. Double peaks appear in Figure 1 around 2*θ* = 23° and 46° as the film thickness exceeds 1,030 nm. The reflections at 22.7° and 46.3° are the (003) *c*-axis orientation and (006) *c*-axis orientation, respectively. The reflections at 23.3° and 47.5° correspond with the *a*-axis alignment of (100) and (200). We use the ratio *I* = *I* (200) / *I* (006) + *I* (200) to evaluate the *a*-axis grains’ volume fraction of the GdBCO film, as shown in Figure 2. In the 200-nm-thick GdBCO film, no (200) peak is observed, so the corresponding ratio *I* is 0% for the thinnest film, which indicates that all the grains grow along the *c*-axis. As the thickness increases to 1,030 and 1,450 nm, the ratio *I* increases to 3.3% and 10.7%, respectively. This illustrates that *a*-axis-oriented grains appear in the 1,030-nm-thick GdBCO film, and the *a*-axis grains’ volume fraction becomes more and more as the thickness comes up to 1,450 nm. When the thickness approaches to 2,100 nm, the volume fraction of *a*-axis grains increases to 31.6%. The *a*-axis grains are film defects that will block current flowing in GdBCO films. They will cause the degradation of *J*_c_[12,13].

**Figure 3:**
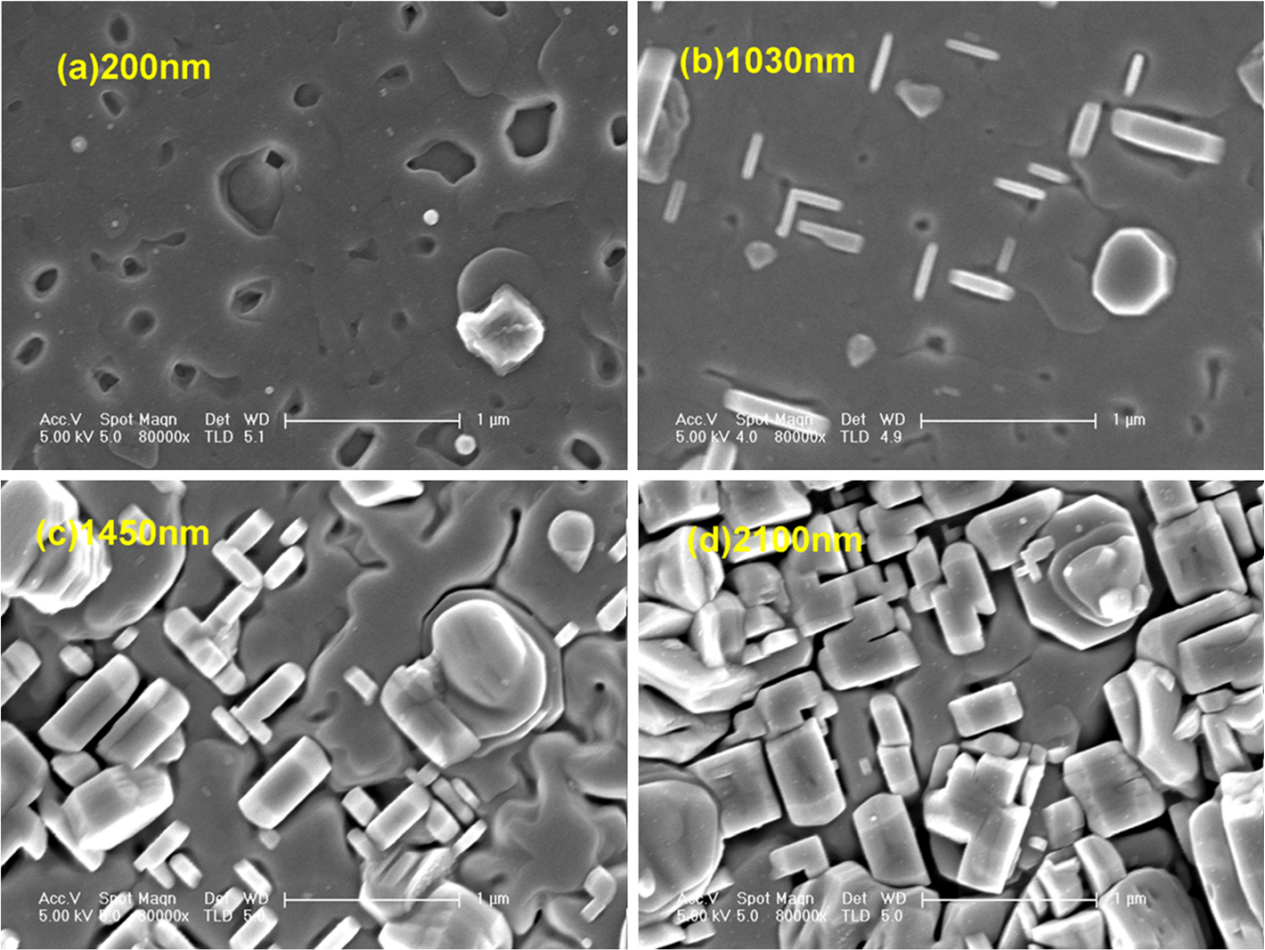
**SEM images of GdBCO films with different thicknesses fabricated under optimized fabrication conditions. (a)** 200 nm. **(b)** 1,030 nm. **(c)** 1,450 nm. **(d)** 2,100 nm.

In order to further look into the development of the microstructure for GdBCO films with various thicknesses, we measure the surface morphologies of the studied GdBCO films by SEM and AFM. Figure 3a,b,c,d shows the SEM images of GdBCO films with the thicknesses of 200, 1,030, 1,450, and 2,100 nm, respectively. For the 200-nm-thick GdBCO film, there are a few pinholes on its surface. The appearance of pinholes for (RE) BCO films was first observed by Low et al. [14] (in their Figure four) by pulsed laser ablation method. They associated the pinholes with stronger oriented grains along the *c*-axis [14]. Tao et al. [15] (by sputtering method, in their Figure seven), Chen et al. [16] (by advanced low-fluorine solution method, in their Figure four), and Vermeir et al. [17] (by fluorine-free water-based sol–gel method, in their Figure five) also reported a similar pinhole appearance. In another series of experiments for GdBCO films deposited with different temperatures, we find that a higher temperature favors the emergence of pinholes while a lower temperature favors a flat film without pinholes. It is well known that for (RE) BCO films, a higher temperature is advantageous for *c*-axis grain growth while a lower temperature is advantageous for *a*-axis grain growth. Therefore, it is believed that the appearance of pinholes for our films indicates stronger oriented grains along the *c*-axis in the film.

As the thickness increases to 1,030 nm, rectangular-shaped outgrowths appear on the film surface. This implies *a*-axis grains of the GdBCO film. At the same time, both the size and number of pinholes become smaller (Figure 3b). The pinholes disappear for samples F1450 and F2100 (Figure 3c,d). The disappearance of pinholes for thicker GdBCO films can be attributed to a temperature decrease effect of top layers for thicker GdBCO films. Because the GdBCO film is a bad thermal conductor, the top layer will not be heated sufficiently. Hence, it is indicated that the disappearance of pinholes for thicker films probably results from a decrease of deposition temperature for the top layer. This explanation accords very well with our above discussion for the appearance of the pinholes in thinner films. The mechanism of the pinholes is still not clear. They will also damage the superconducting performance of the (RE) BCO films because they will decrease the effective supercurrent-carrying cross-sectional area.

From Figure 3c, it can be seen that the size and number of the rectangular-shaped outgrowths become bigger and more when the thickness increases to 1,450 nm. With the thickness increasing to 2,100 nm, the rectangular-shaped outgrowths are overlapped together. Some gaps are left between the grains. This will certainly lower the GdBCO films’ density and decrease the *J*_c_ value with increasing film thickness.

**Figure 4:**
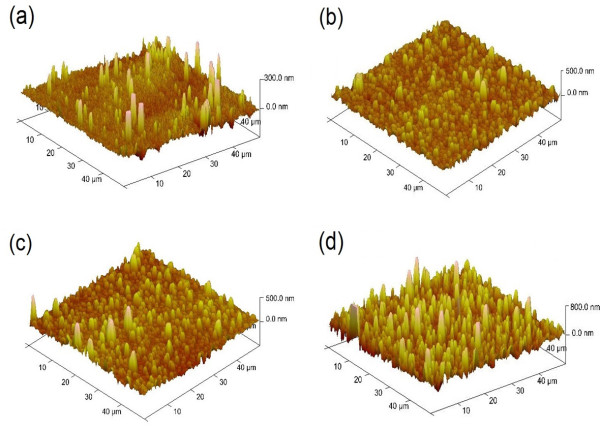
**Surface morphologies of GdBCO films with various thicknesses. (a)** 200 nm. **(b)** 1,030 nm. **(c)** 1,450 nm. **(d)** 2,100 nm.

The surface roughness for our samples is measured by AFM, which is shown in Figure 4. The RMS value for the 200-nm-thick film is 23.6 nm. As the film thickness increases to 1,030 nm, the RMS value is 64.6 nm. For further increase of the film thickness to 1,450 nm, there is a little RMS value increasing from 64.6 to 68.7 nm. It is believed that the appearance of *a*-axis grains for the 1,450-nm-thick film results in a slower increase of the RMS value. It is found that films grown with pure *a*-axis grains at low temperature in another experiment show a rather flat surface morphology. The RMS value goes up to 73.5 nm in the case of the 2,100-nm-thick film. Roughness measurement is in agreement with the observation of SEM (Figure 3). It is believed that the biggest RMS value for the 2,100-nm-thick film arises from the gaps between *a*-axis grains, as shown in Figure 3d.

### Stress analysis by means of the Williamson-Hall method

**Figure 5:**
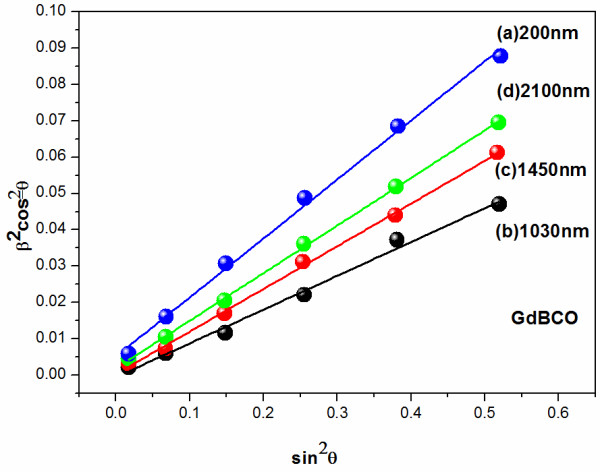
**Williamson-Hall plot for GdBCO films with different thicknesses.** In this image, *β* is the Bragg angle position of each (00*L*) peak. The internal strain *ϵ* can be obtained by the slope of this fitting of the data points.

Up to now, the stress effect for the GdBCO films has not been discussed yet by us. In reality, the Williamson-Hall method is an old and effective method to analyze film internal strain *ϵ* by XRD measurement [18]. The relationship of the internal strain *ϵ* and the integral breadth *β* value of each (00*L*) peak of the GdBCO film is as the expression:

(1) $$16\epsilon^{2}=\frac{\beta^{2}\cos^{2}\theta }{\sin^{2}\theta },$$

where *θ* is the Bragg angle position of each (00*L*) peak, *λ* is the value of X-ray wavelength (*λ* = 1.5418 Å). Figure 5 shows *β*^2^cos^2^*θ* variation as a function of sin^2^*θ* for the GdBCO film with different thicknesses. Using the obtained linear fit slopes in Figure 5, the residual stresses calculated using Equation 1 are 0.101, 0.076, 0.086, and 0.091 for the four GdBCO films, respectively. The corresponding film thicknesses are 200, 1,030, 1,450, and 2,100 nm, respectively. It is concluded that the thinnest film has the highest residual stress while the 1,030-nm-thick film has the lowest residual stress. With further increase of the film thickness, the film residual stresses increase again.

**Figure 6:**
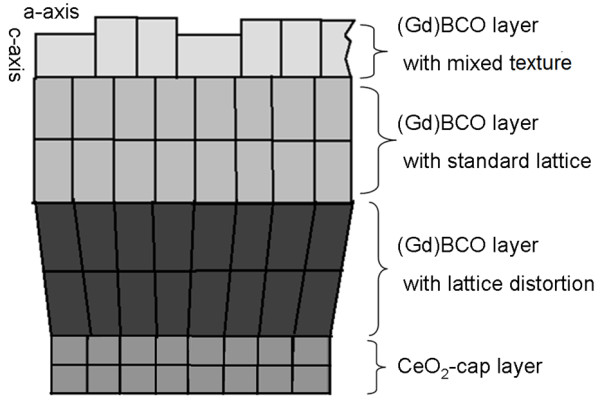
Schematic diagram of possible growth model for thick GdBCO films on CeO_2_/YSZ/CeO_2_-buffered Ni-W substrates.

The Williamson-Hall method has a disadvantage that it cannot make a distinction between compressive stress and tensile stress. To get further insight into the stress behavior of the GdBCO films, more studies are needed. Because the cubic lattice constant of the GdBCO (*a* = 3.831 nm, *b* = 3.893 nm, from JCPDS card no. 82–2292) is bigger than the pseudocubic lattice constant of CeO_2_ (3.826 nm), a big compressive stress may appear at the interface of the substrate and as-grown top film on it, and it will gradually release with the increase of the thickness of the film in order to reduce the compression. In our case, with enhancing film thicknesses from 200 to 1,030 nm, the residual stresses decrease from 0.101 to 0.076. It is indicated that the compressive stress caused by the lattice mismatch of the CeO_2_ cap layer and the above GdBCO film can be released when the film thickness comes up to a certain value such as 1,030 nm. It should be noted that a stress conversion appears at the thickness of 1,030 nm. Tensile stresses occur at one location far away from the CeO_2_ cap layer. Xiong et al. [10] found that the tensile stress appeared when the film thickness reached 1,000 nm. Zeng et al. [11] have reported similar results. Xiong et al. believed that oxygen vacancies were the reason of the tensile stress [10], while Zeng et al. attributed the tensile stress to the more *a*-axis grains and the bigger surface roughness value with increasing thickness of the film [11]. In our case, we believe that the increase of residual stress for thicker films, such as F1450 and F2100, may be due to the increase of *a*-axis grains in the GdBCO film, which will cause the tensile stresses in GBCO film’s (a, b) plane. A possible and simple growth model (shown in Figure 6) considering the lattice change is used to explain the variation of the stress with increasing thickness of the film.

For the thinner GdBCO film, the film grows with lattice distortion, which results in compressive stresses. As the film thickness increases to a critical thickness, such as 1,030 nm, the GdBCO film grows with a standard lattice. Therefore, the compressive stresses are released. With the further increase of the thickness of GdBCO films, *a*-axis grains appear. At the same time, the bigger roughness value for thicker films will lead to tilted GdBCO grains. The two factors result in tensile stress emergence.

### Oxygen content analysis by XPS

**Figure 7:**
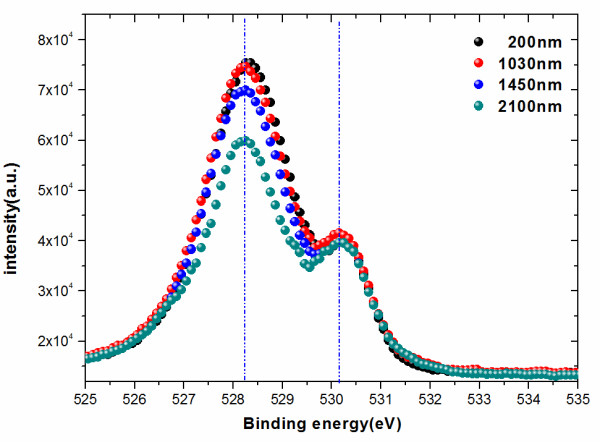
**O 1*s *spectra measured for GdBCO films with different thicknesses.** (black) 200 nm. (red) 1,030 nm. (blue) 1,450 nm. (green) 2,100 nm. The two vertical lines in the image show the two peaks’ positions.

XPS is performed to determine the oxygen content of the studied GdBCO films. The XPS measurement is under slot mode, and the analysis area is 700 × 300 μm^2^. The analysis chamber pressure is less than 5 × 10^−9^ Torr. Generally, only information from the surface of the film (5 to 10 nm) can be examined by XPS measurement. However, all the films are fabricated under the same conditions except for fabrication time. Hence, the XPS measurement of GdBCO films with different thicknesses is equivalent to the XPS depth profiling measurement of one thicker film. The spectra obtained for O 1*s* is shown in Figure 7. The O 1 *s* spectra consist of two peaks. The main peak at *E*_B_ = 528 to 528.5 eV is related with the O characteristic of the CuO_2_ plane and Cu-O chains in superconducting (RE) BCO [19]. The small one at *E*_B_ = 530 to 530.5 eV may be associated with some nonsuperconducting phases [19,20]. It can be seen that the intensity of the two peaks decreases with increasing film thickness from 200 to 2,100 nm. This indicates that there is less oxygen content for the upper layer of the thicker film compared to thinner ones. At the same time, the curve integral area for the four samples decreases as the film thickness increases from 200 to 2,100 nm. This is a direct proof for less oxygen content for the upper layers of the thicker film. The two trends are not obvious between the 200-nm-thick film and the 1,030-nm-thick film. However, when the film thickness increases to 1,450 nm, the two trends become obvious. The above analysis implies that the oxygen contents are insufficient for the upper layers of the thicker film, especially for the film thicker than 1,030 nm.

As mentioned above, the XPS measurement of GdBCO films with different thicknesses is equivalent to the XPS depth profiling measurement of sample F2100. The oxygen content is different for different depth layers for one thick film. For the bottom layer from 0 to about 1,030 nm, the oxygen content almost does not change. For the upper layers from 1,030 to 2,100 nm, the oxygen content reduces. The oxygen deficiency for the upper layers beyond 1,030 nm for thick films may result in bad superconductivity, which will be discussed in the next part.

The outgrowths on the thick films will obviously affect the results of the XPS measurement. The analysis area is 700 × 300 μm^2^, so the area will contain many outgrowths (see Figure 4c,d). The outgrowths will contribute to the signals of XPS measurements. The outgrowths are mainly consisting of *a*-axis GdBCO grains. The oxygen content reduction is accompanied with the emergence of *a*-axis grains for the upper layers of the thick film. It implies that the oxygen deficiency for the upper layers beyond 1,030 nm of thick films mainly results from *a*-axis grain emergence.

### Superconducting performances of GdBCO films

**Figure 8:**
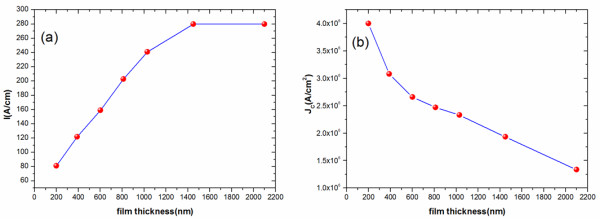
***I***_**c**_** (a) and *J***_**c**_** (b) measurements of GdBCO films with different thicknesses under optimized deposition conditions.**

Figure 8a shows the superconducting current *I*_c_ of the studied GdBCO films. It is found that there is a nearly linear relationship between film thickness and *I*_c_ as the film thickness increases from 200 to 1,030 nm. Several possible factors affect the value of *I*_c_ for our GBCO films: residual stress, surface roughness, *a*-axis grains, and oxygen content. For the films with a thickness between 200 and 1,030 nm, the variations of residual stress and surface roughness do not affect the supercurrent carrying ability because of the nearly linear relationship between film thickness and *I*_c_. It is indicated that the release of residual stress and the increase of surface roughness are not the main factors influencing the superconducting performances. With the film thickness increasing from 1,030 to 1,450 nm, the *I*_c_ value increases more slowly. There is a little change with surface roughness for the two samples. However, much more *a*-axis grains appear in the 1,450-nm-thick film compared with the 1,030-nm-thick film. Apart from these, it is suggested that there is less oxygen content for the upper layers beyond 1,030 nm for samples F1450 and F2100. It is believed that the appearance of much more *a*-axis grains and the less oxygen content for the upper layers of thick films are the two main factors affecting the superconducting performances for samples F1450 and F2100. When the film thickness approaches to 2,100 nm, it is worth noting that there is nearly no supercurrent increase with increasing film thickness from 1,450 to 2,100 nm. The phenomenon is first reported by Foltyn et al. [21]. They attributed it to a porous microstructure of the top layer. In our case, it is found that the gaps between *a*-axis grains will result in porosity inside the top layer. As a result, the porosity inside the film and the gaps on the film surface will block the supercurrent for the 2,100-nm-thick film. Besides, the oxygen deficiency for the upper layer of the thicker film is another factor affecting the superconducting performances. For our GdBCO films, the superconducting performances are subject to three factors: *a*-axis grains, gaps between *a*-axis grains, and oxygen deficiency. The stress and the roughness are not the main factors affecting the superconducting performances. Figure 8b shows the *J*_c_ value of our studied films. It can be seen that the thinnest film, F200, exhibits the highest *J*_c_. The mechanism discussed above cannot explain why F200 has the highest *J*_c_ value. Van der Beek et al. [22] reported that a maximum in *J*_c_ was obtained at a thickness between 100 and 200 nm. This result is similar to our studies. Foltyn et al. [8] attributed the very high *J*_c_ for the thinnest YBCO films to the high density of misfit dislocations near the interface of the substrate and the above YBCO film. We believe that the high-level compressive stresses in F200 leads to the highest *J*_c_ values.

Tao et al. [15] reported the *J*_c_ of YBCO film to be 1.6 × 10^6^ A/cm^2^ at 77 K and self-field with a thickness of 1.2 μm by sputtering method on buffered Ni-5 at.% W substrates. Tran et al. [23] found that the 0.2-μm-thick GdBCO film had the highest *J*_c_ of 3.8 × 10^6^ A/cm^2^ and the *J*_c_ value decreased to 4.2 × 10^5^ A/cm^2^ as the film thickness increased to 0.55 μm. From our results, the *J*_c_ of the 1,450-nm-thick film can achieve as high as 2.0 × 10^6^ A/cm^2^. At the same time, a nearly linear relationship between film thicknesses and *I*_c_ has been found when the film thickness is below 1,030 nm. A linear relationship between thickness and *I*_c_ is very important for achieving high current carrying ability for thick films. Recently, several ways have been developed to solve the thickness effect in (RE) BCO films. Using multilayer technology, Foltyn et al. have achieved *J*_c_ values of up to 4.0 × 10^6^ A/cm^2^ in the film with a thickness of 3.5 μm, at_75 K, self-field on metal substrates [9]. Tran et al. have overcome the rapid decrease of *J*_c_ value by BaSnO_3_ addition in (Gd) BCO films [23]. Feldmann et al. achieved a *J*_c_ (75.6 K, self-field) of 5.2 × 10^6^ A/cm^2^ in a single-layer 2.0-μm-thick YBCO film with BaZrO_3_ (BZO) and Y_2_O_3_ additions [24]. Dürrschnabel et al. obtained the *J*_c_ of (Dy) BCO film to be 1.7 × 10^6^ A/cm^2^ at 77 K and self-field with a thickness of 5.9 μm on inclined substrate-deposited MgO-buffered Hastelloy substrates [25]. These research results are exciting. Our next research work will focus on finding methods to overcome the thickness effect in (RE) BCO films.

## Conclusions

GdBCO films with different thicknesses are prepared on CeO_2_/YSZ/CeO_2_-buffered Ni-W substrates by means of RF sputtering. The stress and microstructure of the GdBCO films with various thicknesses are investigated by XRD, SEM, AFM, and XPS techniques. For the 200-nm-thick film, the highest *J*_c_ value of 4.0 MA/cm^2^ has been obtained. The highest *J*_c_ value is attributed to high-level compressive stresses for the 200-nm-thick film. A nearly linear relationship between *I*_c_ and film thickness is observed as the film thickness increases from 200 to 1,030 nm. It is realized that differences of stress and roughness do not affect the supercurrent carrying ability with increasing film thickness. We find that when the film thickness approaches to a certain value about 1,030 nm, the *a*-axis grains appear at the upper surface. As a result, more and more *a*-axis grains lead to lots of grain gaps, which will certainly reduce the effective supercurrent carrying cross section. In addition, oxygen deficiency is found for upper layers beyond 1,030 nm for F1450 and F2100. It can be understood that the slower increase of *I*_c_ for the 1,450-nm-thick film and no increase of *I*_c_ for the 2,100-nm-thick film are due to *a*-axis grains, gaps between *a*-axis grains, and oxygen deficiency for the upper layers of the thick film.

## Abbreviations

AFM: Atomic force microscopy; CeO2: Cerium oxide; GdBCO: GdBa_2_Cu_3_O_7 − *δ*_; HTS: High-temperature superconducting; Ic: Critical current; Jc: Critical current density; RF sputtering: Radio-frequency magnetron sputtering; RMS: Root mean square; XRD: X-ray diffraction; YSZ: Yttria-stabilized-zirconia.

## Competing interests

The authors declare that they have no competing interests.

## Authors’ contributions

YW participated in the design of the study, carried out the preparation of Ni-W tapes with buffer architectures, carried out the fabrication of GdBCO films, performed the statistical analysis, as well as drafted the manuscript. LL participated in the design of the study and revised the manuscript. DX helped operate the RF magnetron sputtering system. YL participated in the design of the study, provided the theoretical and experimental guidance, and revised the manuscript. All authors read and approved the final manuscript.
